# Biogenic Silver Nanoparticles for Targeted Cancer Therapy and Enhancing Photodynamic Therapy

**DOI:** 10.3390/cells12152012

**Published:** 2023-08-07

**Authors:** Glory Kah, Rahul Chandran, Heidi Abrahamse

**Affiliations:** Laser Research Centre, Faculty of Health Sciences, University of Johannesburg, Johannesburg 2028, South Africa; glorykah26@yahoo.com (G.K.); habrahamse@uj.ac.za (H.A.)

**Keywords:** anticancer, nanotechnology, phytonanotechnology, biogenic silver nanoparticles, photodynamic therapy, toxicity

## Abstract

Different conventional therapeutic procedures are utilized globally to manage cancer cases, yet the mortality rate in patients with cancer remains considerably high. Developments in the field of nanotechnology have included novel therapeutic strategies to deal with cancer. Biogenic (green) metallic silver nanoparticles (AgNPs) obtained using plant-mediated protocols are attractive to researchers exploring cancer treatment. Biogenic AgNPs present advantages, since they are cost-effective, easy to obtain, energy efficient, and less toxic compared to chemically and physically obtained AgNPs. Also, they present excellent anticancer abilities thanks to their unique sizes, shapes, and optical properties. This review provides recent advancements in exploring biogenic AgNPs as a drug or agent for cancer treatment. Thus, great attention was paid to the anticancer efficacy of biogenic AgNPs, their anticancer mechanisms, their efficacy in cancer photodynamic therapy (PDT), their efficacy in targeted cancer therapy, and their toxicity.

## 1. Introduction

The commencement of cancer is provoked by an uncontrolled division of cells, and these cells can then invade nearby normal tissues [1]. Mutations in tumor suppressor genes and proto-oncogenes are often involved in cancer initiation [2]. Cancer remains a leading cause of mortality, and it is expected that its global burden will increase by 2040 with about 28.4 million cases to be recorded [3]. Globally, different conventional treatments are used for cancer treatment by oncologists, including chemotherapy, radiation, and surgery [4]. Yet, most cancers are very resistant to these treatments, leading to a low survival rate in cancer cases [5]. Often, chemotherapy appears as the first option for cancer therapy, and the chemotherapeutic molecules used in this therapy are widely used as primary targets to destroy cancerous cells. However, both healthy and cancerous cells are destroyed by chemotherapeutic molecules since they may be non-target-specific [6,7] These molecules are also noted for inducing acute side effects in patients, and some normal functioning cells, including those in the digestive tract, mouth, reproductive system, hair follicles, and bone marrow blood-forming cells are likely to be injured [6]. Secondary therapeutic methods used in cancer treatment, such as immune and hormone therapy, can also cause severe side effects and abnormalities in patients, such as damage to normal cells and organs, causing a deterioration in the quality of life [8]. Similarly, no current therapy for treating cancer is alluded to as presenting selective blinding to cancerous cells, which leads to unsolicited toxicities and side effects [9]. Moreover, cancer cells are made of cellular and non-cellular components that differ from those of normal cells. These altered cellular components hinder the transportation and delivery of tumor drugs, leading to poor drug bioavailability [4]. Identifying new treatment strategies for the effective management of cancer has also been problematic [7]. The aforementioned pitfalls associated with conventional cancer treatments have motivated the search for more cost-efficient and strongly sensitive molecules that may exercise cell-targeted specificity in cancer treatment [4,10]. This may lessen the burden linked to cancer treatment.

Innovative therapeutic intervention for cancer via nanotechnology suggests metallic silver nanoparticles (AgNPs) as promising nanoproducts for cancer treatment. They are confirmed to have anticancer properties, including the selective obstruction of the respiratory chain in the mitochondria, resulting in reactive oxygen species (ROS) and impairment of DNA [11]. AgNPs are obtained via the transformation of silver ions using nanotechnology into ultra-small materials that are quantified in nanometers (nanoscale) [12]. The transformation of the bulk silver ion to AgNPs via greener or biogenic methods presents advantages over chemical and physical methods. For instance, the utilization of plants in the biogenic production of AgNPs is denoted as being very cost-effective, presents less hazards to humans and the environment, and is easy to perform [13]. Likewise, silver in nanoscale dimensions (AgNPs) is depicted to present new physicochemical properties and can promote unique biological activities [14]. AgNPs have again applications in diagnosis, microelectronics, solar energy conversion, catalysis, water treatment, and photonics [15]. They are also used in cosmetics, hygienic goods, detergent production [12] domestic appliances, and ink-jet printing [1]. The uniqueness of AgNPs equally broadens their exploration in various therapies for their antifungal, antiviral, antibacterial, antiangiogenic, anti-inflammatory, and anticancer properties [14]. Moreover, great antitumor effects of AgNPs have been reported [1], and biogenic AgNPs can ameliorate the anticancer ability of photodynamic therapy (PDT) [16]. Moreover, carryover phytochemicals in biogenic AgNPs can be liberated in cancerous cells due to their acidic microenvironment, and they can aid in augmenting the anticancer efficacy of AgNPs. Taking into consideration the existing knowledge on AgNPs and their anticancer impacts, this review, therefore, focuses on the anticancer effects of biogenic AgNPs with special emphasis on the synthesis, anticancer potential, anticancer mechanisms, effectiveness in cancer PDT, and toxicity. 

## 2. Nanotechnology

Within the last decade, knowledge regarding the tumor microenvironment has also inspired scientists to investigate various nanotechnology methods for cancer treatment and diagnostic purposes [4]. The nanotechnology domain encompasses different interdisciplinary fields such as medicine, biomaterials, and electronics [12]. Rapid development in the era of nanotechnology has led to the exploration of new inventions in medicine and biology [15]. The nanomedicine area focuses on improving the health sector by proposing more efficient procedures for dealing with mortal diseases. Nanotechnology-based applications make use of nanostructures (nanoparticles or nanomaterials), which are produced via nanotechnology techniques such as the synthesis, design, and maneuvering of large structures into nanoparticles [12]. The ISO/TR 18401:2017 (en) describes nanoparticles as materials with lengths ranging from 1 to 100 nm [17]. 

Generally, nanomaterials are categorized into organic, carbon-based, and inorganic. Organic-based nanomaterials such as dendrimers, liposomes, ferritin, and micelles are often exploited as delivery systems for the targeted release of active drug ingredients [18]. Carbon-based nanomaterials such as carbon black, fullerenes, carbon nanotubes, graphene, and carbon nanofibers are wholly arranged with carbon [19]. Inorganic-based nanomaterials such as metal oxide and metallic nanomaterials are told not to have any carbon atoms within their composition [18]. Metallic nanoparticles such as aluminum, cadmium, copper, lead, cobalt, iron, zinc, gold, and silver can present intrinsic properties thanks to their characteristics and size, including pore size, expanded surface, spherical and cylindrical shape, structures (crystalline and amorphous), surface charge density, and color [15,18]. The formulation of nanoparticles with sizes or lengths from 1 to 100 nm has been reported in various research studies and is considered for its biomedical applications [6]. Nanoparticles are exploited in other fields like cosmetics, drug delivery, and therapeutics because of their distinct biological, physical, and chemical qualities, and they may hopefully enforce cancer therapy [12,20]. Nanoparticles are about 10 to 10,000 times smaller in size than cell organelles, making their application favored in extracellular (surface) and intracellular therapeutic and diagnostic procedures [1]. Moreover, the new development in the applications of nanostructures might have encouraged different companies such as Sigma Aldrich, Evonik, BASF, Blue Nano, Blue Nano, Cima Nanotech, Carestream Advanced Materials, PolyIC, Dow Chemical, Saint-Gobain, Advanced Nano Products Co., Ltd., SILVIX Co., Ltd., Nano Silver Manufacturing Sdn Bhd, Ames Goldsmith Corporation, NovaCentrix, Applied Nanotech Holdings, Inc., Creative Technology Solutions Co. Ltd., Bayer MaterialScience AG, NanoMas Technologies, Inc., ras Materials, and Suzhou NanoGrid Technology Co., Ltd. to produce and market nanomaterials [21]. The global market for engineered nanomaterials is about 11.5 million tons, and this is estimated to have a market value of $20 billion a year [21]. Likewise, metallic nanomaterials, particularly AgNPs, are reported to have had steady market growth over the last decade, and the global production of AgNPs is estimated at 500 tons a year [21,22]. AgNPs as well as gold nanoparticles (AuNPs) are the most exploited metallic nanomaterials. They are used in the chemical and biomedical sectors, show anticancer activity, and can act as catalysts [23]. In addition, biologically obtained AgNPs and AuNPs are said to have antimicrobial, antioxidant, and anticancer activity [24]. However, AgNPs has excellent antimicrobial properties compared to other metallic nanoparticles and can act as carriers for chemotherapeutic molecules, and this has strengthened their applications in different sectors [25,26]. AgNPs are also documented as the most commercialized nanomaterials, account for more than 50% of consumer nano-products globally, and are expected to have about 13% market growth from 2016 to 2024. This could be associated with the predominant applications of AgNPs in life science, health care, information technology, electronics, and the food and packaging sectors [27]. Also, advanced products for wound dressing manufactured in the form of composites of ionic silver, such as ActicoatTM, AquacelTM, BactigrasTM, TegadermTM, or Poly Mem SilverTM, have been approved by the Food and Drug Administration (FDA) [26]. Yet the market price for AgNPs seems expensive, as the price is determined by the producing company based on the particle size [21]. This apparently shows that a cost-effective method to produce nanomaterials like AgNPs should be exploited.

The formulation of metallic nanomaterials via the recent year’s research advocates for cost-efficient methods and the utilization of these nanomaterials in highly sensitive applications including clinical diagnosis, molecular biology, and cancer therapy [15]. Metallic nanomaterials like AgNPs are the most utilized nanomaterials due to their impressive functionalities attributed to their unique chemical and physical qualities. AgNPs exhibit stronger effects compared to the bulk ion. Silver is a noble metallic element that is resistant to bacteria and is a promising antibacterial agent [12]. Nonetheless, silver can be engineered and manipulated using new nanotechnology procedures to create new structures with exciting properties [12]. 

## 3. Biogenic AgNPs Synthesis

**Figure 1:**
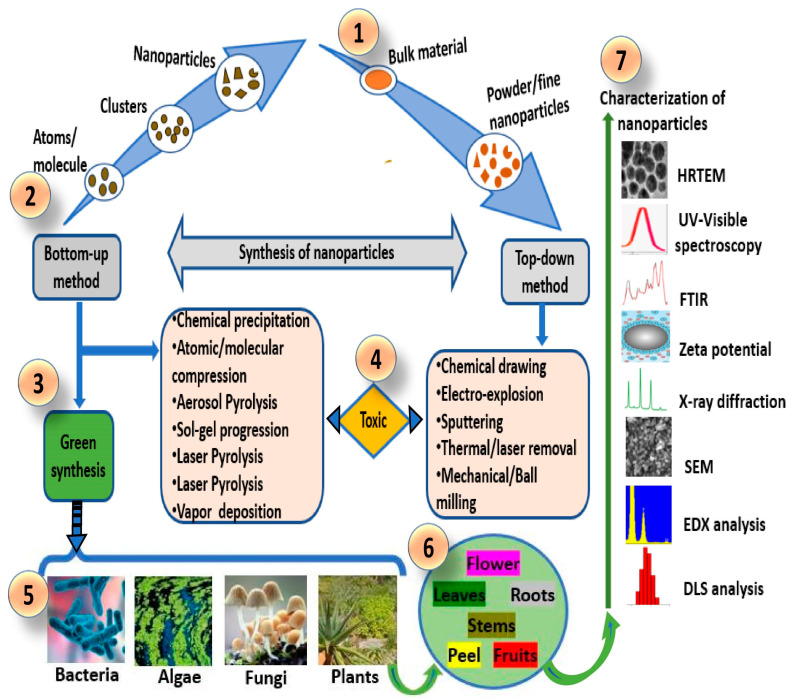
Bottom–up and top–down methods for nanomaterial synthesis. (**1**) Synthesis using bulk material in the top–down method; (**2**) synthesis using atomic structures/molecules in the bottom–up method; (**3**) green synthesis approaches in bottom–up methods; (**4**) toxic method for nanomaterial synthesis in bottom–up and top–down methods using physical and chemical approaches; (**5**) biological sources exploited in bioformulation of biogenic (green) nanomaterials; (**6**) biological plant parts that are used in biogenic nanomaterials synthesis (**7**); characterization techniques to confirm the synthesis of nanomaterials.

Green (biogenic) nanotechnology focuses to engineer nontoxic nanoscale materials by exploring eco-friendly and biological materials while minimizing the energy consumed in the process. Green nanotechnology methods for producing AgNPs involve the bioreduction of the metallic ion (Ag^+^) to the AgNPs (Ag^0^), and this requires an appropriate biological source [13,28]. Functionalized nanomaterials can be produced using green methods via the amalgamation of biological and physicochemical principles [29]. Chemical and physical methods are also exploited for the synthesis of AgNPs. The green synthesis and chemical methods for synthesizing nanostructures are categorized as bottom–up methods, while the physical methods are categorized as top–down methods (Figure 1). 

The bottom–up methods describe the merging of molecules/atoms to formulate nanostructures. On the other hand, top–down methods involve the miniaturization of large materials into fine nanostructures [29,30]. The chemical methods require very expensive chemicals that are often hazardous to humans and the environment. Also, the physical methods require enormous force and energy, which elevates production costs and has a harmful effect on the environment. Nonetheless, biomaterials explored in the green synthesis of nanostructures are said to be superior to those used in chemical and physical methods in numerous ways, including excessive availability of biomass, low cost, and nontoxicity, and handling is very easy and safe [30,31]. Macroscopic or microscopic biomaterials from bacteria, algae, yeasts, seaweeds, fungi, plants (leaves, stem, bark, flower, seedlings, shoots, fruit, roots, twigs, peel, gum, latex, plant secondary metabolites, and essential oils), pods-tissue cultures, and biopolymers are utilized in the synthesis of biogenic nanomaterials or particles [13]. The exploitation of plant biomaterials to synthesize metallic nanomaterials, including AgNPs (phytonanotechnology), offers more advantages than microorganisms, as the latter need specific aseptic conditions in order to maintain pure microbial cultures. The preservation of stabilized cultures is also extremely complex if factors for cell culture such as pH, salinity, and temperature are considered [32,33,34]. Moreover, the phytonanotechnology methods are simple, cost-efficient, and pose no environmental threat. The nanoparticles engineered via this method are generally stable, and the speed of synthesis is relatively fast. Large amounts of nanoparticles of different sizes and shapes, free from contaminants, can be generated from plant sources. The quantity of bioactive phytochemical compounds in plant materials such as proteins, polysaccharides, vitamins, enzymes, phenols, alkaloids, terpenoids, saponins, and tannins determines the size and shape of the nanoparticle, as these compounds naturally accelerate the reduction of bulk materials to the formation of metal ions by dually acting as reducing and stabilizing agents [13,34]. Yet, an enhancement or alteration in reaction conditions including temperature, pH, salt concentration, duration of incubation, and redox conditions can affect the obtainable sizes and shapes of nanomaterials. For example, the size of AgNPs synthesized using plants can be affected by altering the pH. The pH alterations may induce changes in the plant phytochemicals by changing their charge, thus altering the reduction and binding processes during AgNP synthesis [35]. Alterations in pH also influence the zeta potential of the obtained nanoparticles, since changes in ionic strength in the reaction solution will cause changes in the cationic nature of Ag^+^. Also, a temperature increase in the reaction medium will accelerate the reaction rate, which affects the thermal stability of reducing agents and the final yields. Likewise, the obtainable sizes and shapes for AgNPs greatly depend on the proportion of silver nitrate (AgNO_3_) salt to plant biomaterial used in synthesis [36]. 

## 4. Anticancer Efficacy of Biogenic AgNPs 

Generally, plant biomaterials often contain medicinal phytochemicals that can enhance the efficacy of biogenic nanomaterials against different types of microorganisms and cancer cells. In addition, biogenic AgNPs from plant sources have a spotlight feature due to their phytochemical coating, which furnishes them with improved biological activity compared with AgNPs engineered using chemical methods [37,38]. In different studies, plant-mediated AgNPs of different sizes and shapes have been engineered and characterized using various techniques (UV-visible spectroscopy (UV-vis), high-resolution X-ray diffraction (HR-XRD), Fourier transform infrared spectroscopy (FTIR), zeta potential, high-resolution transmission electron microscopy (HR-TEM), energy-dispersive spectroscopy analysis (EDS), field-emission scanning electron microscopy (FE-SEM), dynamic light scattering (DLS), zeta potential (ZP), atomic force microscope (AFM), field-emission transmission electron microscope (FE-TEM), energy-dispersive X-ray analysis (EDAX), photoluminescence (PL), thermogravimetric analysis (TGA), and nanoparticle tracking analysis (NTA)). Different concentrations of these biogenic AgNPs have been showcased in numerous in vitro research studies to exhibit promising anticancer abilities (Table 1).

## 5. Photodynamic Therapy (PDT)

PDT in cancer therapy describes a noninvasive treatment modality that utilizes light of a specific wavelength and a compatible photosensitizing agent (nanoparticles, chemicals, or drugs) to treat various types of cancer. The activation of the photosensitizer (PS) in tumor cells by light irradiation can trigger a reaction with molecular oxygen to produce ROS, which causes cellular damage in diseased cells [65], antineoplastic immunity stimulation, and tumor blood vessel damage [66]. PDT procedures are known to have great therapeutic efficacy and minimal side effects, and they are also less costly compared to cancer conventional therapeutic procedures [67]. Nonetheless, some drawbacks are noted that limit the application of PDT in cancer treatment. For instance, most traditional PSs are hydrophobic, often aggregate, show poor biodistribution, and are not selective or target specific [68,69]. These limitations deleteriously impact the photophysical, biological, and chemical attributes of PSs and thus diminish the effectiveness of PDT [70].

Ideally, an efficient modality for drug (PS) delivery should surmount these drawbacks, and the PSs should be biologically compatible and degradable within the targeted microenvironment of the cells while exhibiting a lesser uptake by healthy cells [65,68]. The inadequate supply of oxygen in most solid tumors (tumor tissue hypoxia) also significantly limits the therapeutic efficacy of PDT as the procedure is oxygen-dependent [71,72]. To overcome the aforementioned PDT limitations, novel photosensitizers are now being developed, including nano-drug systems that can facilitate the target delivery of drugs to the tumor and nano-enzymes that can assist in catalyzing H_2_O_2_ to O_2_, hence improving the oxygen content in tumor tissues [73]. Moreover, metal-based nanostructures can be utilized as photosensitizers, up-conversion tools, and drug delivery vehicles [74]. 

Furthermore, the solubility of therapeutic hydrophobic molecules and PDT drugs can be improved using nanoparticles. This can allow for therapeutic molecules or drugs with appropriate surface properties and sizes to circulate for a longer duration in blood, thus allowing the selective accumulation of the drug in tumors via an enhanced permeability and retention (EPR) effect [75,76]. In fact, nanoparticles are said to be auspicious in cancer therapy due to their therapeutic potential. They can be utilized as delivery vehicles for therapeutic molecules, lone material-based for PDT, and in combination with chemotherapeutic molecules to improve the efficiency of photo-treatment [66,77]. For instance, metallic nanoparticles are naturally biocompatible and may be excreted easily from the body. They can be utilized as therapeutic moieties carriers when conjugated or wrapped with therapeutic moieties. The surface conjugation of metallic nanoparticles with a specific target moiety can modify the metal nanoparticles to target specific cancer cells [69]. 

### Optical Property of AgNPs for Cancer PDT

Porphyrins and silicon phthalocyanines are the most commonly used organic PSs (chromophores) and are reported to have several limitations. These limitations include poor phototability, low molar extinction coefficients, an inability to be stimulated by near-infrared light (NIR), and ineffective enzymatic degradation. This is associated with the fact that the wavelength of light within the UV-Visible spectrum cannot adequately penetrate the tissue depths [78]. However, metallic NPs are affirmed to present numerous advantages compared to organic PSs, such as conjugation efficiency or high loading, slow degradation, high stability, adjustable size, long cycle time, easy surface functionalization, and good optical properties. These attributes make metallic PSs highly biocompatible and able to resist disintegration in biological applications. This can promote tumor targeting and the targeted control delivery of PSs [78,79]. Metallic nanoparticles present specific chemical (improved catalytic activity) and physical (such as fluorescence enhancement and plasmon resonance) properties [80], making them explorable in PDT [16]. For instance, metallic nanoparticles including AgNPs are known to strongly react when in contact with light, and this is known to be a surface plasmon resonance (SPR) phenomenon [81,82]. 

However, only a few metals, such as lithium, copper, aluminum, palladium, platinum, gold, and silver, can act within the visible light region as potent plasmonic nanomaterials. The nanostructure formation, chemical stability, plasmonic resonance, and cost of each of the listed metals can influence their disadvantages or advantages in plasmonic applications. For instance, silver is known to have the strongest resonance, and its spectrum covers a broad range (from 300 to 1200 nm). Following silver are gold and copper, with localized surface plasmon resonance (LSPR) excitation wavelengths correspondingly above 500 and 600 nm. Nevertheless, the utilization of copper in biological applications is greatly hindered by its toxicity and instability. Palladium and platinum are the most expensive plasmonic nanomaterials and have the weakest resonance, which makes them not suitable for large-scale applications. Aluminum is mostly effective in the UV region, while lithium is extremely reactive, making its manipulation at the nanoscale level very difficult [83]. 

The above-mentioned attributes for metallic plasmonic nanomaterials thus indicate that silver can be a suitable metallic chromophore. The SPR phenomenon triggered via the interaction of AgNPs with specific light is comparatively more efficient than that produced by known inorganic and organic chromophore compounds [81,82]. The restriction by large-density circulating electrons with smaller dimensions relative to the dielectric function (at a specific frequency) and the mean free path for metallic silver are responsible for the strong interaction of AgNP with light, which then stimulates the unique SPR phenomenon. The shape and size of NPs, as well as the dielectric function within the medium, greatly determine the resonance and frequency strength [81,82]. In addition, the interaction cross-section for light and AgNPs depends on the electric field generated by photons, which may extend to about 10 times greater than the AgNPs geometric cross-section. This makes some nanostructures, like AgNPs, able to interact with rays of light (photons) that may not be incident directly upon them [84]. Fascinating results can also be obtained by modifying certain optical properties of AgNPs. For instance, the absorption spectrum of AgNPs can be tunable to the region of near-infrared absorption by carefully optimizing the conditions (such as pH, temperature, salt concentration, and time) for AgNPs synthesis. This can help eliminate tissue autofluorescence interference, resulting in nanomaterials that are promising for deep-tissue imaging and for targeting tumors [85]. A study exploring AgNPs and AuNPs as chromophores indicated their colors could be tunable from 400 to 750 nm. Parameters or conditions such as the morphologies (nanospheres, circular nanodisks, triangular nanoplates, and nanocubes of silver), structures (solid, hollow colloid), and controllable composition (silver/gold alloy nanospheres) were linked to the tunable change. Tunable SPR bands were produced if the mentioned parameters were altered. Also, the decrease in the nanoparticle’s symmetry resulted in an increase in the number of SPR peaks [86]. The aforementioned described property of AgNPs with light (unique optic property) thus facilitates the exploration of AgNPs in noninvasive techniques, including dark field microscopy (for tracking inspection and cellular uptake evaluation) and PDT [1,84].

## 6. Mechanisms of Biogenic AgNPs and in Combination with PDT

The cytotoxicity effects of AgNPs on mammalian cells are reported to be triggered via different types of mechanisms, such as the production of reactive oxygen species (ROS) and free radicals, damage to the cell membrane, which is attributed to direct contact with AgNPs, DNA replication impairment, disruption of cellular-dependent energy processes due to free silver ion uptake [87] and stimulation of apoptosis [12]. For instance, a comparative study analyzing the effects of AgNPs and AgNO_3_ on Chang liver cells found that AgNPs could promote the production of ROS, suppress glutathione reduction, and cause membrane oxidation, protein carboxylation, and DNA damage. Also, a major damaging effect of AgNPs was linked to an increase in 8-oxoguanine levels [88,89]. Another study compared the effects of AgNPs and Ag^+^ on human T-lymphocyte immortalized cells (Jurkat T). Similar levels of ROS were induced in the cells by both AgNPs and Ag^+^ within the first period of exposure, whereas an increase in ROS was noticed after 24 h for Jurkat T cells treated with AgNPs only. This could be due to the slow liberation of silver ions by AgNPs into the cell, leading to oxidative stress [90]. The AgNPs exposure was suggested to activate p38 mitogen-activated protein kinase (p38 MAPK) via nuclear factor-kappa B (NF-κB) and nuclear factor-erythroid-2-related factor-2 (Nrf-2) pathways and subsequently cause cell cycle arrest, DNA damage, and apoptosis. Moreover, the alkaline comet assay (for direct DNA damage) and the formamidopyrimidine–glycosylase FPG–comet assay (for oxidative DNA damage) were used for DNA repair and damage studies [90]. The findings suggest that the direct DNA damage induced by AgNPs cannot be completely repaired because of the presence of silver ions, which are slowly being released by internalized AgNPs. Meanwhile, oxidative damage may be achieved via the cellular repair system [91,92].

In vitro studies indicate that AgNPs can penetrate cells via the process of endocytosis, and the localization of the AgNPs in the cells can be established based on the appearance of a cytoplasmic perinuclear space and an endolysosomal unit [93,94]. Kalishwaralal et al. [95] indicated that AgNPs can alter the proper functioning of vascular endothelial growth factor (VEGF). VEGF is also referred to as the vascular permeability factor and is a mitogen in endothelial cells. VEGF upregulation is stimulated by hypoxia in diseased cells and holds a fundamental role in the angiogenesis of tumors [95]. Thus, the alteration of VEGF by AgNPs supports its anticancer potential, which suggests AgNPs can be utilized as an alternative therapeutic method for cancer and also in angiogenesis inhibition therapy [95,96]. Angiogenesis can arise from existing blood vessels and is vital for processes involved in embryogenesis and homeostasis, such as the regeneration and repair of impaired tissues. The deregulation of angiogenesis can occur under certain disease conditions. However, malignant diseases can cause the angiogenesis process to remain active due to increased stimulation by angiogenesis factors, including tumor angiogenesis factors (TAFs). These factors are secreted in response to the oxygen and nutritional needs of cancerous cells, hence their progression and growth [97]. Angiogenesis can also promote metastasis, as the vascular network that is developed via angiogenesis may not only function to provide nutrients to malignant cells but also provide an escape route for these cells to move into the circulation [98]. 

AgNPs can infiltrate the mitochondria to produce ROS by altering cell respiration processes [95]. AgNPs’ increased toxicity is linked to ROS production [99]. The internalization of AgNPs in cells is followed by their intracellular degradation. Silver ions are then released to impair the functioning of the mitochondria. ROS resulting as by-products from the electron transport chain can cause damage to the mitochondria and peroxidation of proteins and lipid elements, eventually leading to apoptosis [100]. AgNPs themselves can also induce ROS production [101]. A Fenton-like reaction showed that AgNPs dispersed in an acidic milieu containing hydrogen peroxide (a stimulated environment) can induce ROS-like hydroxyl radicals [102]. Hydrogen peroxide at a low concentration within the cell can speed up AgNPs dissolution, leading to much oxidative stress [101]. A study exploring five kinds of triangular-shaped AgNPs (tAgNPs) with particle sizes ranging between 25 and 50 nm and satisfactory dispersion revealed that the tAgNPs in vitro treatment triggered cellular apoptosis via ROS production and increased activity of caspase 3. The tAgNPs also led to a decrease in the proliferation and viability of SKOV3 cells, G0/G1 phase cell cycle arrest, and inhibition in the expression of proliferation-associated factors and proteins (cyclins) [103]. Cyclins proteins are responsible for the activation of cyclin-dependent kinases (CDK) during the cell cycle [104]. These CDKs are the main regulatory enzymes responsible for regulating cell proliferation by controlling the three principal checkpoints (G0/1, 1, and 2) involved in the cell cycle process. The cell cycle stages are consolidated into five phases (G (0, 1, 2), S, and M-phases) and are controlled by the three checkpoints. The levels in CDK function to regulate the development from one phase to the next [105]. Unregulated cell proliferation can be promoted by oncogene activation and by suppressor tumor genes (such as p53) inactivation. The overexpression of these genes can lead to an arrest of the cell cycle or make the cells circumvent their cellular checkpoints [106]. Normally, cells can trigger cellular mechanisms that can block DNA-damaged cells from moving into the cell cycle’s G1 or G2 stages. However, an elevation in p53 levels can be induced thanks to the presence of DNA-damaged cells. These p53 genes may then function as transcription factors by regulating cell growth [107]. The p53 genes can enhance the upregulation of p21 proteins and also induce the transcription of proteins like BH3 in the pro-apoptotic phase. To prevent cells from going via the various cell cycle phases, the p21 protein can attach to CDKs and cyclins, thus hindering their oncogenic action at the G1, 2, and S cell cycle phases [105,107].

A study by Jia et al. [108] on the effect of AgNPs on human colon cancerous cells (HCT116) and normal colon cells (NCM-460) conveyed that as the AgNPs’ concentrations increased, the cellular activities in both colon cell lines were reduced, while the intracellular ROS was increased. The Western blot and RT-qPCR assays revealed that AgNPs can activate the increase in p38 protein phosphorylation thresholds in both cells and also enhance the expression of Bax and p53. The down-expression of Bcl-2 was noted; this caused an increase in the proportion of Bax/Bcl-2 and the stimulation of p21, leading to the accelerated death of cells. The AgNPs at low concentrations presented no toxic impact on both cell lines (HCT-116 and NCM-460 cells), while the utilization of higher concentrations (>15 µg/mL) led to oxidative damage [108]. However, green AgNPs may trigger some of the alluded mechanisms above and even other mechanisms since they can be naturally capped with bioactive organic compounds during their synthesis. 

### 6.1. Mechanisms of Biogenic AgNPs as Lone Molecules for Cancer

AgNPs that are produced using a biological (green) route are reported to trigger ROS production, which can cause cell death. Also, the produced ROS may strike pathways for signal transduction and cause cell apoptosis. Hydrogen peroxide triggered due to the presence of AgNPs can interfere with mitochondrial membrane potential to impair respiration signals [109]. The entry of AgNPs into cells has led to NF-κB and tumor necrosis factor-alpha (TNF-α) stimulation and a reduction in levels of glutathione (GSH). Elevations in levels of superoxide radicals can affect the transmembrane potential of the mitochondria to interrupt transduction pathway signals, leading to cell death [110]. The reduction in GSH and elevated levels of ROS could cause key cellular components to be impaired, including protein carbonylation, lipid membrane peroxidation, and DNA fragmentation [87]. Bio-mediated AgNPs are also proposed to trigger apoptosis via various mechanisms, such as sub-G1 phase cell cycle arrest, dependent pathways for mitochondrial and caspases, caspase-3 and p53 protein stimulation, VEGF activities, ROS production and cellular equilibrium disruption, the pH-dependent liberation of Ag^0^, and the targeted killing of cancer cells [90]. The death of cancerous cells or cancer cell’s selective killing can also link to the concentration of free silver ions released in the cells. However, the release of Ag^0^ in cancerous and normal cells is greatly determined by the pH of the medium and the electrostatic differences in these cells [36]. For instance, excessive silver ions released from biogenic AgNPs at low pH (acidic pH) were affirmed to cause the selective killing of targeted cancer cells [36]. 

Furthermore, biological AgNPs can stimulate the upregulation of p53 protein, which is followed by cell toxicity or cell death [11]. Studies have linked biologically mediated AgNPs with the upregulation of p53 and caspase-3 [111]. A study that utilized the sqRT-PCR method for determining the mRNA expression threshold of apoptotic gene markers including p53, Bax, Bcl-2, and p21 established that the exposure of MCF-7 cells to *Rosa damascenes* AgNPs led to elevated apoptosis. The p53 gene expression in the MCF-7 cells was upregulated by 1.6 fold, while the p21 mRNA expression was significantly upregulated by about 2.3 fold. Also, a remarkable upregulation in the mRNA expression for Bax was noted, while the mRNA expression for Bcl-2 was downregulated by 65% when compared to cells that were not treated. This resulted in an elevated Bax/Bcl-2 ratio [112]. The treatment of A549 cells with *Coptis chinensis* biogenic AgNPs induced the upregulation of pro-apoptotic proteins Bak and Bax, while the anti-apoptotic Bcl-XL and Bcl-2 proteins were downregulated [113]. Pro-apoptotic proteins such as Bak and Bax are often involved in initiating or stimulating apoptosis, whereas the Bcl-XL and Bcl-2 categories of proteins function by suppressing apoptosis (anti-apoptotic) [114]. The downregulation of the Bcl-2 pathway by biogenic AgNPs also played a vital role in stimulating cancer cell death via NF-κB activation [115].

Furthermore, a study by Banerjee et al. [40] demonstrated the impact of *Mentha arvensis* AgNPs against MCF-7 cancerous cells, and the expression of cleaved caspase 9, p53, P21, PARP1, Bax, and Bcl-2 was determined at various time intervals by exploring the Western blot technique. The expression of cleaved caspase 9, p53, P21, PARP1, and Bax was observed after the AgNPs treatment of cells, whereas a down-expression of Bcl-2 was noted [40]. The upregulation of p53 and P21 proteins could cause a delay in the cell cycle and induction of apoptosis [116], and RARP1 is reported to be activated at the intermediate phase of apoptosis [40]. A study exploring the cytotoxic effect of *Rubus fairholmianus* biomediated AgNPs recorded elevations in ROS production, cytotoxicity, cytochrome c release, caspase 3/7 activity, nuclear damage, mitochondrial membrane potential depolarization, and a decreased proliferation of cells. Also, these green AgNPs induce a significant expression of proteins including caspase 3, p53, and Bax [117]. Alterations in mitochondrial membrane potential that are induced thanks to the cellular uptake of biogenic AgNPs can activate caspases (such as caspase 3 and 9) to cause cellular apoptosis. The activation of c-Jun NH2 terminal kinase (JNK) by this nanoparticle can stimulate the production of apoptotic bodies and the formation of DNA breaks, which could cause an arrest in the cell cycle [87]. A study by Manikandan et al. [118] using *Rosa indica*-mediated AgNPs on HCT 15 cells confirmed the down-expression of Bcl-2 as well as the up-expression of Bax, and caspases 3 and 9. They indicate that the biogenic AgNPs induced death in HCT 15 cells via the mitochondrial-dependent pathway that was activated due to caspases 3 and 9 up-expression [118]. In addition, the mitochondria might be the main site for biogenic AgNPs to trigger ROS production, which then stimulates pathways for intrinsic apoptosis within the mitochondria and hence induces cell death via the caspase pathway [118]. This implies that the mitochondria could function as a signaling central point during apoptosis, and damage to the mitochondrial integrity may be inhibited or stimulated via various apoptotic regulators. 

AgNPs that were formulated using the seed extract of *Putranjiva roxburghii Wall* (PJAgNPs) were validated to have damaging effects on the DNA of various cell lines, including MDA-MB 231 (resistant breast carcinoma), PANC-1 (pancreatic carcinoma), and HCT-116 (colon carcinoma). The IC_50_ concentration of PJAgNPs causes DNA fragmentation in all the cell lines [119]. The accumulated AgNPs in the cells at the time of DNA fragmentation can severely impact the DNA and dividing cells by triggering DNA dose-dependent damage, chromosomal segregation errors, chromosomal aberrations, micronuclei formation, and sister chromatid exchanges [119,120]. Cell DNA damage and subsequent apoptosis/necrosis are associated with excessive oxidative stress and ROS induced by the AgNPs in the cancer cell [121,122]. Apoptotic stimulation can also be generated via the cytotoxic effect of biogenic AgNPs because of an increase in cell numbers at the sub-G1 phase of the cell cycle. A correlation was established between an enriched cancer cell population at the sub-G1 phase and the pro-apoptotic caspase-3 protease that was stimulated due to the presence of AgNPs, thus leading to apoptosis [40]. Glucose-capped AgNPs were demonstrated to hamper the cell cycle in HeLa cells by stopping the S and G2/M phases, causing an increase in cell number at the sub-G1 phase and a decrease in mitotic index [123]. AgNPs from the seed extract of *Swietenia macrophylla* (SM-AgNPs) induced an arrest of the cell cycle at the S-phase in A549 cells. The arrest was suggestive of DNA damage, and the associated defective cells could not enter the phase of mitosis; thus, any further progression in cycle activity can result in cellular apoptosis. 

**Figure 2:**
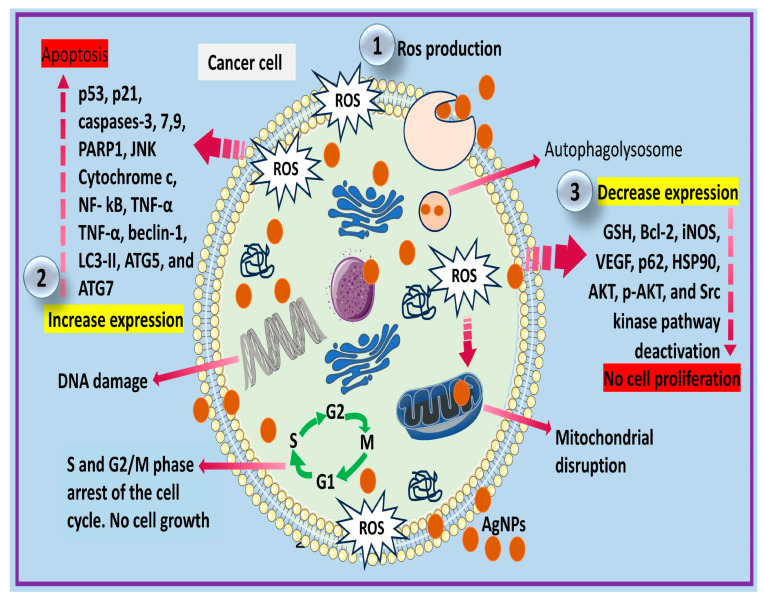
Possible mechanism induced by biogenic AgNPs in cancer cells. (**1**) Ros production is stimulated by released of biogenic AgNPs in cancerous cells; (**2**) Ros will stimulate the up-expression of apoptosis proteins and enzymes (including p53, p21, caspases-3, 7,9, PARP1, JNK, cytochrome c, NF-kB, TNF-α, TNF-α, beclin-1, LC3-II, ATG5, and ATG7) leading apoptosis; (**3**) decrease the expression of GSH, Bcl-2, iNOS, VEGF, p62, HSP90, AKT, p-AKT, and the Src kinase pathway deactivation can inhibit the proliferation of cancerous cells. These increases and decreases in the expression of the various proteins and enzymes triggered by the cytotoxic species (Ros) causes DNA damage, mitochondrial disruption, and cell cycle arrest. Damage can also be triggered via AgNPs autophagolysosomes formation.

Biogenic AgNPs are revealed to have antiangiogenic effects by hindering cell proliferation, and this was provoked by VEGF. The entry of biogenic AgNPs into the cell by the Src-dependent pathway can cause VEGF obstruction and also stimulate an interleukin-1 beta (1L-1β) form of vascular permeability via the Src kinase pathway deactivation [87,124]. The antiangiogenic and anti-metastasis effects of *Azadirachta indica*-mediated AgNPs were amplified by the down-expression of iNOS (nitric oxide synthase) and VEGF (angiogenesis-related genes) [97]. The induced down-expression of iNOS by biogenic AgNPs leads to the downregulation of NOS activities. This causes a reduction in the available proangiogenic factors generated by cells. The interaction of iNOS and VEGF can also form the NO–VEGF complex, which creates a target for anticancer molecules to inhibit angiogenesis, thus lowering the progression and growth of cancerous cells [97,125,126]. Biogenic AgNPs can also degrade cells by autophagy [127]. The release of AgNPs in cancerous cells may trigger cell death via the accumulation of autophagolysosomes [127,128]. For instance, biogenic AgNPs embedded in exopolysaccharide (AgNPs-EPS) were confirmed to exert an autophagic cell death mechanism. The fluorescence microscopy image of SKBR3 cells treated with AgNPs-EPS showed autophagolysosomes (bright punctate dots) in the cytoplasm. The Western blot analysis revealed the up-expression of autophagic markers including beclin-1, LC3-II, ATG5, and ATG7, whereas P62, HSP90, AKT, and p-AKT were down-regulated [129]. The aforementioned mechanisms of biogenic AgNPs are illustrated in Figure 2.

### 6.2. Mechanism of Biogenic AgNPs in Combination with PDT

Experimental evidence indicates that AgNPs can be employed in cancer PDT as lone material-based molecules (PS), in combination with other PS, or in nanocomposite forms. For instance, findings from an experimental study that utilized AgNPs for mediating PDT revealed that the irradiation of AgNPs at 635 nm reduced cell proliferation and viability and triggered apoptosis in both MCF7 and A549 cancerous cells. However, the AgNPs showed a much lower cytotoxic effect on A549 compared to MCF7 cells. This signifies that various forms of cancerous cells can respond differently to identical forms of metallic AgNPs [1]. Cell imaging and PDT studies of engineered nanocomposites of silver (porphyrin-loaded mercaptosuccinic acid-capped AgNPs nanoparticle (POR-MSA-AgNPs)) against A375 cancerous cells had satisfactory output. Although the nanocomposite at a 5 μM concentration was affirmed to have a nontoxic behavior on the A375 cells, excellent fluorescence images were observed at this concentration. This made the researchers recommend POR-MSA-AgNPs as a promising PDT probe [130]. Likewise, a nanocomposite with AgNPs (hypocrellin B (HB) and nanosilver loaded poly lactide-co-glycolide (NBS-NPs)) significantly improved ROS in PDT. The NBS-NPs also showed a concentration- and time-dependent phototoxic effect on lung cancer cells (A549) [131]. The PDT photoactivation of curcumin and AgNPs loaded in hydrogels (chitosan and chondroitin sulfate) hydrogel) led to significant decreases in Caco-2 cells and increased singlet oxygen [132]. 

**Figure 3:**
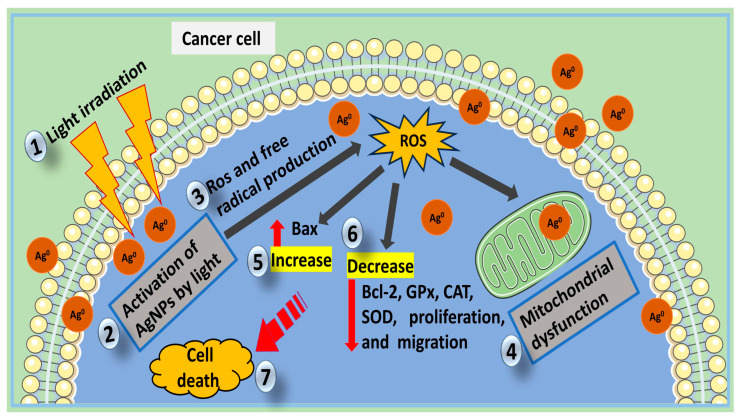
Anticancer mechanism of biogenic AgNPs in cancer PDT. (**1**) Light irradiation from an appropriate source will (**2**); activate biogenic capped AgNPs in cancerous cells (**3**), this will lead to free radical and Ros production (**4**); mitochondrial Ros cause damage. (**5**) Decrease in Bcl-2, GPx, CAT, SOD, cell proliferation, and migration (**6**); and increase in Bax stimulated by Ros will cause the up-expression apoptotic signal to finally (**7**) apoptotic cell death.

Moreover, recent experimental evidence indicates that biogenic AgNPs can be used to improve the efficacy of PDT [16]. Nonetheless, it seems that less interest is paid by researchers to the exploration of biogenic AgNPs in PDT; hence, only a few studies have reported the mechanisms (Figure 3) that stimulate biogenic AgNPs in PDT. An in vitro study that utilized biogenic AgNPs as drugs in PDT affirmed the efficacy of the treatment against breast cancer cells (MCF7 cells). The treatment led to an increase in intracellular production of ROS and a decrease in antioxidant enzymes including GSH, glutathione peroxidase (GPx), catalase (CAT), and superoxide dismutase (SOD). The treatment also inhibited the growth, viability, and migration of MCF7 cells at IC_50_ (10 mg/mL) via the production of free radicals in the cells [16]. Another study utilizing AgNPs and PDT combined on MDA-MB-468 cancer cells affirmed a threefold increase in intercellular ROS in treated cells compared to the control [133]. ROS production is correlated to mitochondrial phosphorylation, and this ROS can be involved in mitochondrial pro-apoptotic processes in tumor cells, leading to apoptotic cell damage [134]. Response processes to mitochondrial ROS production often include the activation of cell death proteins (especially the pro-apoptotic proteins’ upregulation) and suppression of anti-apoptotic proteins [135]. *Cynara scolymus* AgNPs combined with PDT exhibited effective anticancer potential against MCF7 cells via mitochondrial apoptosis. The AgNPs and PDT combination treatment stimulated the pathways for intrinsic apoptosis via the upregulation of Bax (pro-apoptosis protein) and downregulation of Bcl-2 (anti-apoptotic protein) [16]. Some of the aforementioned mechanisms of biogenic AgNPs in PDT are similar to those triggered biogenic AgNPs (lone-base molecule) in cancer. Nonetheless, more research on the anticancer effects of biogenic AgNPs amalgamated with PDT would help to better understand the associated mechanisms. Figure 3 illustrates the induced light-stimulated mechanism of biogenic AgNPs in cancer PDT. 

## 7. Biogenic AgNPs in Cancer-Targeted Therapy 

Conventional treatment methods for cancer, which include surgery, radiotherapy, and chemotherapy, are linked with numerous limitations, including unpredictable side effects, drug toxicity, non-specificity, and drug resistance issues [136]. Chemotherapy is the first line of treatment for most cancers. However, chemotherapeutic agents are not cell-specific (target-specific) and end up also killing healthy cells. Also, the medication (e.g., doxorubicin, cisplatin, bleomycin, and daunorubicin) used in this therapy presents several disadvantages, including high toxicity, ineffectiveness, resistance susceptibility, and high cost [137,138]. AgNPs can overcome these limitations by decreasing the side effects and improving the therapeutic efficacy of the method. AgNPs have the distinguishing feature of being capable of crossing biological barriers and can also be used for the targeted release of drugs [136,139]. They are noted as drug carriers that can be efficiently conjugated with anticancer drugs because of their distinctive characteristics, such as low side effects, enhanced SPR, and a large surface area [140,141]. Moreover, the conjugation of drugs with AgNPs can be achieved via bottom–up and top–down techniques [142]. These techniques solicit strategies like entrapment, encapsulation, and attachment of the active drug to the nanoparticle surface, such that the conjugated product can be enhanced to be biocompatible, stable, and present minimal toxicity [143,144]. In addition, modifying the surface structure of AgNPs is greatly important, since it helps reduce their toxicity, prevents aggregation, and enhances their potential to target particular cells [145]. Gali-Muhtasib et al. [146] allude that an effective nanocarrier for the targeted delivery of anticancer should meet the following prerequisites: (i) has an affinity and can conjugate with the anticancer drug; (ii) can exclusively liberate the drug within its target site; (iii) the anticancer drug–nanoparticle complex must remain stable in serum; (iv) degradation of the nanoparticle should be safe for the organism [146]. 

AgNPs are now viewed as an alternative treatment strategy for cancer, since they can passively or actively target tumor cells, thereby making these particles considered drug delivery systems (DDSs) [147]. A number of events are considered in the passive targeting of tumors by nanoparticles. A faulty fenestrated vasculature is often formed by tumors that contain big gaps (about 100 to 800 nm). The size of nanoparticles can determine if they may cross these gaps. Small nanoparticles can cross the gaps and be deposited closer to the tumor, which minimizes the exposure of normal cells to these nanoparticles. This consequently decreases the adverse effects of nanoparticles on normal cells [146]. However, the deposition of the active drug at the targeted sites can increase the drug’s therapeutic efficacy. Receptors involved with endocytosis can then facilitate the uptake of the drug into the intracellular space. This shows that this type of active targeting may require molecular recognition. Nonetheless, techniques for optimizing nanomaterials like biogenic AgNPs have been suggested where the particle surface is functionalized with specific target molecules or coated with biocompatible molecules or biodegradable polymers [147]. The utilization of AgNPs coupled with other anticancer drugs may also enable a synergistic effect, allowing for a reduction in the dosage of anticancer drugs. This helps reduce the toxicity of anticancer drugs on normal cells and possibly their side effects [139]

The cytotoxic activities of drugs can be enhanced when the drug is incorporated with AgNPs [138], and various in vitro studies confirmed the anticancer efficacy of commercialized pharmaceutical anticancer drugs (e.g., doxorubicin, epirubicin, alendronate, methotrexate, paclitaxel, folic acid, and gemcitabine) is greatly improved when these drugs are coupled with AgNPs [148,149,150,151,152,153].

Moreover, various studies have demonstrated that biogenic can be exploited as DDSs via conjugation or coupling with anticancer drugs. For instance, studies have established that biogenic AgNPs could be utilized as molecules in DDSs. Biogenic AgNPs formulated using seed extracts of *Setaria verticillata* were successfully loaded with daunorubicin (DNR) and doxorubicin (DOX) (hydrophilic anticancer drugs). The loading efficiency for DNR-AgNPs was 40.25% and that for DOX-AgNPs was 80.50%, showcasing DNR-AgNPs and DOX-AgNPs as novel DDSs [143]. The cellular delivery of a drug molecule via the process of endocytosis may also depend on the size of the nanomaterial. Spherical-shaped AgNPs biosynthesized using *Aerva javanica* extract and conjugated with gefitinib (an anticancer drug) were analyzed using scanning transmission electron microscopy (STEM), and the observed images revealed the presence of nanoparticles with a mean size of 5.7 nm. MCF-7 cells were treated with the conjugate (gefitinib-AgNPs), and a significant reduction in viable cells was noted when compared to MCF-7 cells treated with gefitinib alone. Gefitinib delivery using AgNPs helped augment its efficacy and decrease its side effects [154]. Also, studies by Palai et al. [155] successfully functionalized *Azadirachta indica*-mediated AgNPs into a nanocarrier. The obtained nanocarrier (amino-PEGylated silver-decorated graphene nanocomposites (amion-NGO-AgNPs-PEG)) was utilized for loading the anticancer drug DOX. An enhanced drug-loading capacity of 218% was recorded, and the pH-responsive regulated release of DOX was effective, indicating that the nanocarrier (NGO-AgNPs-PEG) was promising as an anticancer drug DDSs. In vitro cytotoxicity analysis using HaCaT cell lines showed that the functionalized PEGylated-nanographene oxide (NGO-PEG) that was loaded with DOX had a more damaging impact on cancer cells than normal cells when compared with the free DOX treatment. Similarly, elevated cytotoxicity was noticed in Hela cells that were exposed to DOX-loaded NGO-AgNPs-PEG compared to the conjugated NGO-DOX. The authors evoke that an efficient target release or delivery of an anticancer drug within the acidic microenvironment of cancerous cells can promote elevated therapeutic efficiency compared to pure nanographene oxide (NGO). The NGO-AgNPs-PEG was proposed as a biocompatible nanocarrier that may be exploited in the targeted and regulated delivery of anticancer drugs and in theranostic nanoplatforms [155]. In addition, AgNPs from the *Eucalyptus procera* aqueous extract were efficiently loaded with imatinib (IMAB-AgNPs). The IMAB-AgNPs exhibited cytotoxic effects on MCF-7 cells, which were noted to be dose-dependent. The IC_50_ values for IMAB-AgNPs, IMAB, and AgNPs were 1,69, 3.02, and 9.63 um, respectively. The expression of apoptosis genes, including Bax and Bcl-2, was investigated using a real-time PCR procedure, and the results revealed that IMAB-AgNPs could trigger the expression of apoptosis proteins [156]. 

The illustrated in vitro experimental studies above greatly show that AgNPs can be used in targeted cancer therapy cancer. Nonetheless, AgNPs is reported as not being extensively exploited in DDSs due to drawbacks regarding their stability and toxicity [139].

## 8. Toxicity of Biogenic AgNPs 

The Trojan horse effect is proposed as a mechanism to illustrate the toxicity of AgNPs in cells [157]. It hypothesizes that if AgNPs smaller than 40 nm traverse the cellular membrane, then once in the cells, the AgNPs will continuously liberate Ag^+^ in the cell. This continued release of Ag^+^ from AgNPs can result in lipid peroxidation [158]. Also, the Ag^+^ ions can anchor to cells in the host and are absorbed before they reach vital organelles in normal cells [22,158]. Yet, a cellular defense response can be mediated by normal cells, where the reductase enzyme is secreted to lessen the damaging effects of Ag^+^. The AgNPs and the liberated Ag^+^ can be finally engulfed and carried outside the cell. The deposit of AgNPs and their ionic form (Ag^+^) in normal cells is regarded as the starting point for toxicity and hazardous effects. The Ag^+^ ions can react with negatively charged atoms, including nitrogen and oxygen within vital organelles (mitochondrion, DNA) and with the thiol functional group of enzymes and proteins. This can interfere with normal cell growth, which eventually leads to cell death [22].

Nonetheless, the toxicity of AgNPs in humans can be initiated via external (contact with the skin) or internal (inhalation or ingestion) exposure [26,159]. The skin is well known to be semipermeable and may not allow nanoparticles to simply penetrate through. For instance, a study by Kokura et al. [160] confirmed that treating the skin with AgNPs led to significant preservation effects against various fungi and bacteria, while no AgNPs were noted to penetrate into the skin. Also, treating HaCat keratinocytes with 0.002 to 0.02 ppm of AgNPs and UVB irradiation resulted in a non-significant effect [160]. Nonetheless, Lu et al. [91] documented that the uptake of AgNPs via the skin keratinocytes depends on the nanoparticle’s shape and size and duration of incubation. Hence, AgNPs with rod and spherical shapes can infiltrate the skin and their cellular uptake was influenced by the incubation time [91]. AgNPs can also infiltrate the skin, especially if the skin is damaged or compromised [161]. This could be concerning, as knowledge of the mechanism of AgNPs in skin infections seems limited [26]. Likewise, the production, disposal, or washing of the nanoparticles can lead to environmental exposures. This can result in particles being inhaled by humans [162]. The inhaled nanoparticles can be transported and subsequently deposited in a non-uniform manner, and this can be influenced by many factors such as age, pulmonary function, structure of the airway, flow rate, and size of the nanoparticle [162]. AgNPs with a diameter lower than 0.1 μm are reported to deeply infiltrate the alveolar by diffusion, and this can make their clearance mechanism take a much longer period [159,163]. This can trigger severe pathophysiological effects due to long periods of interactions between AgNPs and normal tissues [159]. The infiltration of the alveolar–capillary barrier by AgNPs is confirmed to cause damage to the alveolar epithelial layer [159,164]. Moreover, exposure to AgNPs via inhalation can also end up in oral exposure, as the particles move past the mucociliary escalator and are cleared into the gastrointestinal tract (GIT). When in contact with the mucus layer in the GIT, the NPs are translocated into the circulation and consequently cross the epithelium into various organs. The uptake of NPs that are smaller than 100 nm can mainly occur in epithelial cells via endocytosis [159,165]. AgNPs within the enterocytes can stimulate oxidative stress, inflammation, and DNA damage [159].

However, the toxicity of AgNPs can be influenced by factors including particle size, shape, dose, coating, modifications in the surface structure, and cell type [166,167]. These factors should be carefully examined when investigating the toxicity of AgNPs to ensure the viability and effectiveness of the test [167]. It is reported that AgNPs at diluted concentrations may not harm humans but can kill bacteria, viruses, and many other eukaryotic organisms [14]. An in vivo study on the effects of orally administered AgNPs to ICR mice over a period of six weeks revealed that the AgNPs with small size led to efficient dissemination to different organs, including the liver, brain, and kidneys. No AgNPs were observed in the tissue of ICR mice administered with AgNPs of larger size (323 nm). Considerable increases in the threshold of transforming growth factors (TGFs) were noted in the groups treated with small-sized AgNPs (22, 42, and 71 nm of AgNPs), while no change was noticed in the group treated with 323 nm of AgNPs. The B-cell distribution also increased in the group treated with small-sized AgNPs, and no change was observed in the group treated with 323 nm AgNPs [167]. This may be due to the fact that small-sized AgNPs can easily distribute in the target organ, which can cause organ damage [168]. In addition, AgNPs with small sizes are noted to induce higher toxicity than large-sized particles [169]. Another study investigated the dose effect of AgNPs, where the repeated exposure of mice to oral doses of AgNPs was monitored for 28 days. The findings indicated a dose-dependent increase in cytokines [167]. 

Tiwari et al. [170] studied a sixty-day-long exposure of female Wistar rats to AgNPs at concentrations of 50 and 200 ppm (Lowest Observed Adverse Effect Level (LOAEL) dose). The long exposure resulted in renal ultrastructural damage, renal inflammation, and cell survival factor expression, which trigger necrotic renal cell death [170]. The toxicity of AgNPs to baby organs is alluded to as being dose-dependent, and much damage to organs is correlated with higher doses of AgNPs. Long periods of repeated exposure to a small dose of AgNPs can result in their accumulation in the body. This can promote organ impairment, pathological damage to related organs, and chronic toxicity. Thus, human exposure to AgNPs must be minimized, and the dosage of AgNPs should be chosen carefully to minimize daily life toxicity. 

Moreover, in vivo studies have indicated that nanoparticles, including AgNP, can cause chronic and acute toxicity [170,171,172]. The bioavailability of silver ions was the main toxicity-causative agent in zebrafish embryos [173]. AgNPs synthesized via chemical methods are noted to cause high in vivo genotoxicity and cytotoxicity compared to biogenic AgNPs [174]. This insinuates that biogenic AgNPs can be less toxic and biocompatible than chemically obtained AgNPs. However, the intraperitoneal injection of male Wistar rats with various doses of biogenic AgNPs (50, 100, 200, and 400 ppm) for over 21 days was studied by Tarbalia et al. [175]. Important changes to the rat’s organ coefficient and baby weight were observed after 21 days. The doses of the biogenic AgNPs greater than 50 ppm led to impairments in memory, anxiety, and alterations in the hippocampus redox status, kidney, spleen, and liver. The levels of the lipophilic fluorescent products (oxidative stress markers) were elevated in the tissues of all treated animals compared to the control group [175]. Also, oral administration of different doses (0.5, 5, and 10 mg/kg) of *Psidium guajava*-mediated AgNPs to male Wistar rats for over 14 days led to a minimal elevation of hippocampus and cortex oxidative stress factors (glutathione, nitric oxide, and malondialdehyde). The biogenic AgNPs trigger a dose-dependent reduction in acetylcholinesterase (AchE) activity, and the levels of monoamine neurotransmitters (norepinephrine NE and 5-hydroxytryptamine 5H-T) were also decreased. The neurons’ cellular membrane structures were greatly altered due to the biogenic AgNP treatments. However, the impact of the biogenic AgNPs at 0.5 and 5.0 mg/kg was significantly lower compared to the effect induced at 10 mg/kg (the highest concentration). The authors confirmed that the cytotoxic oxidative changes induced by the biogenic AgNPs were minimal. This was due to the availability of capping, biocompatible, and enhancing molecules on the synthesized biogenic AgNPs [176].

However, clinical therapeutic applications of biogenic silver seem to be lacking. Yet, it is maintained that AgNPs may be toxic to different systems, including the skin, respiratory system, kidneys, eyes, immunological system, and hepatobiliary system [177]. The toxic effects of AgNPs in the development of target therapeutic procedures to overcome cancer, antibiotic-resistance infections, and other diseases are desirable. Nonetheless, the destruction of healthy normal cells should be avoided in targeted therapy [35]. 

## 9. Future Prospects

The exploration of biogenic AgNPs represents an emerging area for research with numerous potent activities. These nanomaterials show good biological activity to target and destroy devastating diseases. They are more toxic to cancer cells than normal cells, which makes them promising for future applications in cancer treatment. Yet, the biological activity of AgNPs can be influenced by intrinsic parameters such as surface charge, shape, and size [114]. This indicates that complete pharmacokinetics and pharmacodynamics profiling studies could be piloted to better understand the biocompatibility, side effects, toxicity, and mechanism of biogenic.

Different techniques developed for the production of AgNPs have led to various applications in medicine [10,12]. Likewise, different studies have biologically produced AgNPs and successfully exploited them for anticancer in vitro studies [40,41,42,43]. However, it seems there is no specified optimum protocol for the development and synthesis of biogenic AgNPs that can be utilized in non-invasive cancer therapy and for the targeted delivery of cancer drugs. Hence, future studies in this regard may lead to the bioproduction of AgNPs that will be exploited in cancer treatment in clinical settings. 

## 10. Conclusions

Globally, cancer remains a major cause of death despite the existence of different conventional treatment strategies. The conventional treatment for cancer is often non-target-specific and costly. This has led to severe side effects and low survival rates in patients with cancer. Developments in nanomedicine recommend biogenic synthesis AgNPs as therapeutic molecules for cancer because of their non-toxic nature, low cost, and biomass availability. Biogenic AgNPs as lone molecules display significant anticancer capability in vitro. Also, the unique physical optic attribute of AgNPs makes them explorable as photosensitizers for cancer PDT. In addition, AgNPs synthesized using natural sources can serve as cost-efficient PS carriers in targeted PDT. Remarkable anticancer effects of biogenic AgNPs in the mediation of PDT have been achieved in vitro studies, yet very few studies have exploited biogenic AgNPs in PDT. This implies that biogenic AgNPs could be utilized as therapeutic anticancer target molecules. Nonetheless, the clinical therapeutic application of biogenic AgNPs as anticancer molecules and agents to mediate PDT seems to be lacking. Also, the toxicity of biogenic AgNPs in humans, as noted in the review, seems to be an extrapolation from in vitro studies or from in vivo animal models. This implies more clinical research is needed to determine the potential anticancer and toxic effects of biogenic AgNPs in humans. 

## Figures and Tables

**Table 1:** Biogenic AgNPs against human cancerous cell lines.

Plant	Part Used	Human Cancer Cell Lines	IC_50_ Values	AgNPs Size (nm) and Shape	Possible Reducing and Capping Agents	Reference
*Dysosma pleiantha*	Rhizomes	AGS cells, MDA-MB-231, and breast cancer cells (MDA-MB-453)	7.14 µM (for AGS), 33.521 µM (for MDA-MB-231), and 36.25 µM (for MDA-MB-453)	76 (spherical)	Carbohydrates, amino acids, and reducing sugars	[15]
*Detarium microcarpum*	Leaves	Cervical cancer cells (HeLa) and PANC-1 cells	84 µg/mL (for PANC-1) and 31.5 µg/mL (for HeLa)	84 (spherical)	Polyphenols, alcohol, carbonyl, and aromatic compounds	[31]
*Artemisia marschalliana*	Aerial parts	Gastric adenocarcinoma (AGS)	21.05 µg/mL	5–50 (spherical)	Phenolic acids and flavonoids	[39]
*Mentha arvensis*	Leaves	Breast cancer cells (MCF-7 and MDA-MB-231)	6.25 μg/mL	4–9 (spherical)	Alcohol, proteins, polyols, aliphatic amine, and alkyl halide	[40]
*Annona squmosa* L.	Fruit	Prostate adenocarcinoma (PC-3)	1.7 ± 0.4 µg/mL	6.63 (spherical)	Phenolic acids, flavonoids, and aromatic compounds	[41]
*Annona Glabra* L.	Fruit	PC-3, ovary adenocarcinoma (SKOV3)	2.4 ± 0.3 (for PC3) and 2.8 ± 0.23 µg/mL (for SKOV3)	7.11 (spherical)	Polyphenols	[41]
*Achillea biebersteinii*	Flowers	MCF-7 cells	20 µg/mL	10–40 (spherical and pentagonal)	Protein and phenolic compounds	[42]
*Tussilago farfara*	Sesquiterpenoids in flower bud	Pancreas ductal adenocarcinoma (PANC-1) cells, AGS, and colorectal adenocarcinoma (HT-29) cells	338.0 μM (for AGS), 275.3 μM (for HT-29), and 166.1 μM (for PANC-1)	13.57 ± 3.26 (spherical)		[43]
*Cleome viscosa* L.	Fruit	Lung adenocarcinoma (A549) and ovarian teratocarcinoma (PA-1) cell lines	28 mg/mL (for A549) and 30 mg/mL (for PA-1)	5–30 (spherical and irregular)	Phenolic compounds, alkaloids, amino acids, tannins, and carbohydrates	[44]
*Potentilla fulgens*	Roots	MCF-7 and human glioblastoma cancer (U-87)	4.91 mg/mL (for MCF-7) and 8.23 mg/mL (for U-87)	10–15 (spherical)	Amino acids, phenolic, flavonoid, and terpenoids	[45]
*Memecylon umbellatum Burm F.*	4-N-methyl benzoic acid (plant derivative)	MCF-7	42.19 mg/mL	7–22 (spherical)	Phenolic derivative (4-N-methyl benzoic acid)	[46]
*Alternanthera sessilis*	Leaves	PC-3 cells	6.85 μg/mL	30–50 (spherical)	Proteins	[47]
*Solanum muricatum*	Leaves	HeLa cells	37.5 µg/mL	20–80 (irregularly)	Flavonoids	[48]
*Cymodocea serrulata*	Leaves	HeLa cells	34.5 μg/mL	17–29 (spherical)	Alcohols, phenols, proteins, alkenes, alkyl halides, ketones, isothiocyanates, and isocyanates	[49]
*Diospyros malabarica*	Fruit	Human primary glioblastoma (U87-MG) cell line	58.63 ± 5.74 μg/mL.	8–28 (spherical)	Polyphenols, proteins, amino acids, peptides, and alkynes	[50]
*Stigmaphyllon ovatum*	Leaves	HeLa cells	9.1 × 10^−9^ µM	24 (spherical)		[51]
*Artocarpus lakoocha*	Fruit	PC-3	30.62 µg/mL	6.6–25 (spherical)	Phenolic, flavonoids, terpenoids, polysaccharides, enzymes, alkaloids, amino acids, alcoholic, and protein compounds	[52]
*Cucumis sativus*	Fruit	PA-1 cells	49.71 μg/mL.	11.12–39 (spherical)	phenolic, and proteins	[53]
*Satureja Rechingeri Jamzad*	Leaves	AGS cells	4.84 μg/mL	62 ± 1 (spherical)	Phenolic, alcohols, and proteins	[54]
*Punica granatum*	Leaves	HeLa cells	100 μg/mL	41.69–69.61 (spherical)	Polyphenols, and flavonoids	[55]
*Punica granatum*	Pell	MDA-MB-231 cells	72.314 µg/mL.	15–30 (spheroidal)		[56]
*Beta vulgaris*	Roots	MCF7, A549, and Hep-2 cell line (pharynx Hep-2)	47.6 μg/mL (for MCF), 48.2 μg/mL (A549) and 47.1 μg/mL (for Hep-2)	5–20 (spherical)	Alcohol, phenols, amine, and aromatic compounds	[57]
*Cucumis prophetarum*	Leaves	A549, MDA-MB-231, HepG-2, and MCF-7	105.8 μg/mL (for A549), 81.1 μg/mL (for MDA-MB-231), 94.2 μg/mL (for HepG-2), and 65.6 μg/mL (for MCF-7)	30–50 (polymorphic shapes; with some ellipsoidal and irregularly granulated)	Tannins, alkaloids, triterpenoids, saponins, phenols, and steroids	[58]
*Lantana camara*	Leaves	A549 and MCF-7 cell lines	49.52 g/mL (for A549) and 46.67 g/mL (for MCF-7)	10–50 (irregular)	Hydroxyl and carbonyl compounds	[59]
*Perilla frutescens*	Leaves	Prostate adenocarcinoma (LNCaP) and colon carcinoma (COLO-205)	24.33 μg/mL (for LNCaP) and 39.28 μg/mL (for COLO-205)	20–50, various shapes (spherical, rod, rhombic, and triangle)	Flavonoids, phenolic triterpenoids, and glycosides components	[60]
*Ginkgo biloba*	Leaves	Cervical carcinoma cell lines (HeLa and SiHa cells)	3 μg/mL for both cell lines	40 (spherical)		[61]
*Derris trifoliata*	Seeds	A549 cells	100 μg/mL	16.92 ± 7 (spherical)	Flavonoids, phenolic, saponins, and proteins	[62]
*Elephantopus scaber*	Leaves	MCF-7, A549, oral squamous cell carcinoma (SCC-40), and colon carcinoma (COLO-205) cell lines	GI_50_ < 10 µg/mL for all the cell lines	59 (spherical)	Phenolic and amino acids	[63]
*Alpinia officinarum*	Rhizome	MCF-7, human small cell lung cancer (H69AR), and Human prostate cancer (DU-145) cells lines	52.4 ± 0.6 μg/mL (for MCF-7), 44.11 ± 1.2μg/mL (for H69AR) and 36.1 ± 2.2 μg/mL (for DU-145)	2.5 and 45.3 (spherical)		[64]

## Data Availability

Not applicable.
